# The Prickly Solution to Metabolic Syndrome: A Multitarget View on the *Opuntia ficus-indica* Fruit Phytocomplex

**DOI:** 10.3390/nu18071157

**Published:** 2026-04-03

**Authors:** Cristina Russo, Sofia Surdo, Maria Stella Valle, Lucia Malaguarnera

**Affiliations:** 1 Section of Pathology, Department of Biomedical and Biotechnological Sciences, School of Medicine, University of Catania, 95123 Catania, Italy; cristina.russo@unict.it; 2 School of Osteopathy, Italian Center for the Study of Osteopathy (CSDOI), 95124 Catania, Italy; s.surdo98@yahoo.com; 3 Laboratory of Neuro-Biomechanics, Section of Physiology, Department of Biomedical and Biotechnological Sciences, University of Catania, 95123 Catania, Italy; m.valle@unict.it

**Keywords:** *Opuntia ficus-indica*, nutraceuticals, metabolic syndrome, insulin resistance, oxidative stress, inflammation

## Abstract

Metabolic syndrome (MetS) is a multifactorial cardiometabolic condition characterized by insulin resistance, visceral adiposity, dyslipidemia, hypertension, and chronic low-grade inflammation, collectively increasing the risk of type 2 diabetes mellitus, non-alcoholic fatty liver disease, and cardiovascular disease. Growing interest has focused on plant-derived dietary strategies capable of targeting multiple pathogenic pathways simultaneously. *Opuntia ficus-indica* fruits (OFIF) represent a complex food matrix containing betalains, polyphenols, carotenoids, soluble fiber, functional amino acids, vitamins, and minerals. Experimental evidence suggests that these constituents interact with key molecular networks implicated in MetS pathophysiology, including redox-sensitive pathways (NRF2), inflammatory signaling (NF-κB), energy-sensing regulators (AMPK), and lipid metabolism proliferator-activated receptor alpha (PPAR-α) dependent mechanisms. Preclinical studies consistently report associations with improvements in oxidative stress, inflammatory markers, hepatic steatosis, and glucose homeostasis following OFIF supplementation. However, human evidence remains limited by small sample size, short intervention duration, and variability in compositional standardization. This narrative review adopts a systems-level perspective to integrate mechanistic, preclinical, and early clinical evidence in the context of metabolic syndrome pathophysiology, while critically addressing translational gaps, compositional variability, and current limitations in human validation.

## 1. Introduction

Metabolic syndrome (MetS) represents an escalating global health challenge, characterized by a constellation of interrelated metabolic abnormalities, including central obesity, insulin resistance (IR), dyslipidemia, hypertension, and impaired glucose tolerance. These interconnected disturbances substantially increase the risk of type 2 diabetes mellitus (T2DM), atherosclerosis, and cardiovascular disease (CVD) [1]. According to the International Diabetes Federation, approximately 25% of the global adult population is affected by MetS, with prevalence projected to rise in parallel with urbanization, suboptimal dietary patterns, and sedentary lifestyles [2].

IR constitutes the core pathophysiological feature of MetS and is closely associated with chronic low-grade inflammation and oxidative stress arising from dysfunctional adipose tissue expansion and hepatic metabolic overload [3]. This altered metabolic environment is characterized by elevated circulating levels of pro-inflammatory cytokines, including tumor necrosis factor-α (TNF-α) and interleukin-6 (IL-6), reduced antioxidant defenses, and mitochondrial dysfunction, all of which impair insulin signaling in skeletal muscle, liver, and adipose tissue [4]. The interaction between inflammatory transcription factors such as nuclear factor kappa B (NF-κB) and redox-sensitive regulators, including nuclear factor erythroid 2-related factor 2 (NRF2), has emerged as a central molecular axis contributing to MetS progression [5].

Given the multifactorial nature of MetS, increasing attention has been directed toward plant-derived functional foods as complementary dietary strategies for cardiometabolic risk modulation. Bioactive-rich foods capable of influencing oxidative stress, inflammatory signaling, hepatic lipid accumulation, adipose tissue function, glucose metabolism, and gut microbiota composition are being investigated for their potential supportive role in MetS management. Among these, *Opuntia ficus-indica* fruits (OFIF) have attracted growing interest due to their complex phytochemical matrix and nutritional profile [6].

Experimental and preliminary clinical studies suggest that OFIF-derived bioactive compounds may exert multi-organ effects by targeting interconnected redox and inflammatory pathways implicated in MetS, including NRF2-dependent antioxidant responses and NF-κB–mediated cytokine signaling, together with regulators of insulin sensitivity and lipid metabolism [7]. However, translational confirmation is still evolving and heterogeneous, underscoring the need for further well-designed randomized trials. Importantly, this review does not aim to provide a systematic aggregation of evidence, but rather to propose a systems-level conceptual framework integrating OFIF bioactives within the complex pathophysiological network of metabolic syndrome, characterized by interconnected redox imbalance, chronic low-grade inflammation, and metabolic dysregulation. By critically examining mechanistic evidence alongside early clinical observations, this work seeks to identify key translational gaps, sources of variability, and priorities for future research.

### Literature Approach and Scope

This review was designed as a narrative, mechanistic synthesis rather than a systematic review. To enhance transparency and minimize potential selection bias, literature retrieval was conducted using PubMed, Scopus, and Web of Science databases, with no formal PRISMA workflow applied. Search terms included combinations of “Opuntia ficus-indica”, “metabolic syndrome”, “oxidative stress”, “inflammation”, “polyphenols”, “betalains”, and “gut microbiota”. Priority was given to recent mechanistic studies, preclinical models, and available human data relevant to metabolic regulation. Studies were selected based on conceptual relevance to redox–inflammatory–metabolic pathways rather than predefined inclusion/exclusion criteria. This approach reflects the objective of providing an integrative, hypothesis-generating framework rather than a quantitative evidence synthesis.

## 2. Pathophysiology of Metabolic Syndrome

MetS can be conceptualized as a maladaptive systems-level condition in which chronic nutrient excess reshapes metabolic communication across organs. Beyond isolated alterations in glucose or lipid handling, it reflects disrupted inter-organ crosstalk involving adipose tissue, liver, skeletal muscle, and the gut, ultimately leading to loss of metabolic flexibility and reduced capacity to maintain homeostatic resilience under nutritional stress [8].

### 2.1. IR, Redox Imbalance, and Inflammatory Crosstalk

IR represents the central metabolic defect in MetS and is characterized by impaired insulin-mediated glucose uptake in skeletal muscle, reduced glycogen synthesis, and increased hepatic gluconeogenesis [9]. In obesity, adipose tissue expansion promotes adipocyte hypertrophy, local hypoxia, and endoplasmic reticulum stress, leading to recruitment of immune cells, particularly pro-inflammatory M1 macrophages [10]. M1 macrophages accumulate around dying adipocytes, forming crown-like structures (CLSs), and secrete cytokines such as IL-1β, IL-6, and TNF-α [11,12].

At the molecular level, inflammatory signaling promotes activation of stress-responsive kinases, including IκB kinase β (IKKβ) and c-Jun *N*-terminal kinase (JNK), which induce inhibitory serine phosphorylation of insulin receptor substrates (IRS-1/2), thereby attenuating Phosphoinositide 3-kinase-Protein Kinase B (PI3K-AKT) signaling and subsequent glucose transporter 4 (GLUT4) translocation in insulin-sensitive tissues [13]. Activation of the NOD-like receptor protein 3 (NLRP3) inflammasome further exacerbates this process via caspase-1-mediated IL-1β secretion, contributing to insulin resistance and chronic inflammation [14]. The resulting impairment of insulin signaling leads to systemic glucose dysregulation and compensatory hyperinsulinemia [15]. Concurrently, hepatic IR fails to suppress gluconeogenesis and dysregulates lipid synthesis, promoting metabolic inflexibility [16]. In parallel, mitochondrial substrate overload in adipocytes and hepatocytes enhances electron leakage from the respiratory chain, leading to excessive generation of reactive oxygen species (ROS) and reactive nitrogen species (RNS) [17]. Under physiological conditions, NRF2 orchestrates the transcription of antioxidant and cytoprotective enzymes, including heme oxygenase-1 (HO-1), superoxide dismutase (SOD), glutathione peroxidases (GPx), and NAD(P)H:quinone acceptor oxidoreductase 1 (NQO1) [18]. However, when antioxidant defenses are overwhelmed, oxidative stress induces lipid peroxidation, protein oxidation, and DNA damage, thereby amplifying inflammatory signaling and contributing to metabolic dysfunction in obesity and related metabolic disorders [19].

**Figure 1:**
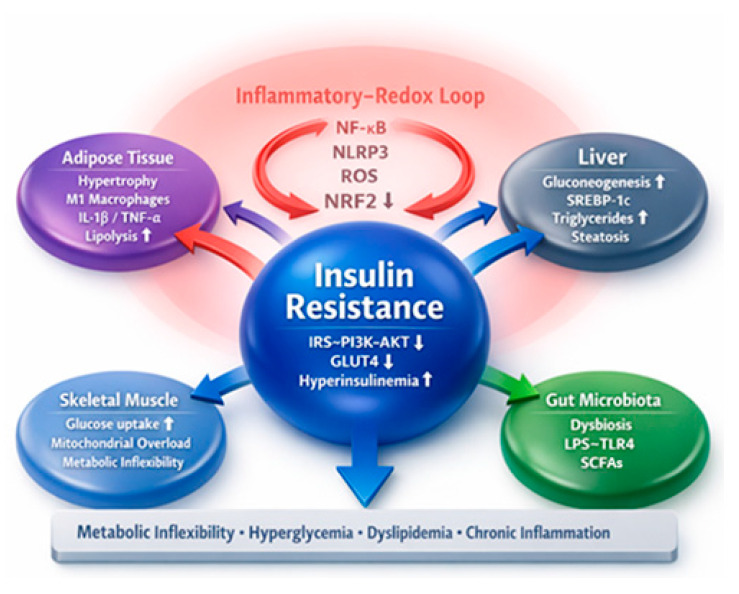
Pathophysiological network underlying insulin resistance in metabolic syndrome. IR represents the central node of MetS, resulting from coordinated dysfunction in adipose tissue, liver, skeletal muscle, and gut microbiota. Activation of the inflammatory redox loop, driven by NF-κB, NLRP3, and excess ROS with impaired NRF2 signaling, promotes systemic inflammation and oxidative stress. These alterations impair IRS-PI3K-AKT signaling, reduce GLUT4 translocation, and lead to hyperinsulinemia, hyperglycemia, dyslipidemia, and metabolic inflexibility. Gut-derived LPS-TLR4 signaling further amplifies inflammation, reinforcing the progression of MetS. Arrows indicate the direction and functional impact of interactions among tissues and pathways. *Abbreviations*: IR, insulin resistance; MetS, metabolic syndrome; NF-κB, nuclear factor kappa B; NLRP3, NLR family pyrin domain containing 3; ROS, reactive oxygen species; NRF2, nuclear factor erythroid 2–related factor 2; IRS, insulin receptor substrate; PI3K, phosphoinositide 3-kinase; AKT, protein kinase B; GLUT4, glucose transporter type 4; LPS, lipopolysaccharide; TLR4, Toll-like receptor 4; SCFAs, short-chain fatty acids.

Excess ROS reinforces inflammatory transcriptional programs through reciprocal modulation of NF-κB and NRF2. In obesity and metabolic dysfunction, impaired NRF2 activation contributes to adipose tissue inflammation and systemic IR [20]. In the liver, defective NRF2 signaling exacerbates oxidative stress, mitochondrial dysfunction, and lipid accumulation, promoting progression toward metabolic dysfunction-associated steatotic liver disease [21,22] (Figure 1). Collectively, disruption of NRF2-mediated cytoprotection establishes a pathogenic loop linking adipose dysfunction, ectopic lipid deposition, and systemic metabolic deterioration.

### 2.2. Hepatic and Adipose Tissue Dysfunction

Systemic IR enhances adipose tissue lipolysis, increasing circulating free fatty acids and hepatic lipid uptake [23]. This metabolic overflow stimulates de novo lipogenesis via SREBP-1c activation, leading to triglyceride accumulation and hepatic steatosis [24]. Lipotoxic intermediates further induce endoplasmic reticulum stress and inflammatory signaling, promoting non-alcoholic fatty liver disease (NAFLD) progression toward nonalcoholic steatohepatitis (NASH) [25]. Concomitantly, adipose tissue undergoes endocrine remodeling. Reduced adiponectin impairs AMPK-mediated insulin-sensitizing effects, whereas elevated leptin, resistin, and pro-inflammatory adipokines sustain systemic metabolic inflammation [26]. The bidirectional interaction between hepatic lipid accumulation and adipose dysfunction further amplifies metabolic derangement [27].

### 2.3. Gut Microbiota and Metabolic Endotoxemia

Dysbiosis, which is a condition due to alterations in gut microbiota composition, has emerged as a key contributor to MetS pathogenesis. Diets rich in saturated fats and refined carbohydrates disrupt microbial diversity, impair tight junction integrity, and increase intestinal permeability, facilitating systemic translocation of lipopolysaccharide (LPS). This condition, termed metabolic endotoxemia, activates Toll-like receptor 4 (TLR4)-dependent inflammatory cascades that exacerbate hepatic and adipose IR [28].

Beyond barrier disruption, gut microbiota-derived metabolites, including short-chain fatty acids (SCFAs), secondary bile acids, and indole derivatives, exert systemic metabolic effects. SCFAs such as butyrate and propionate enhance epithelial integrity, regulate gluconeogenesis, modulate mitochondrial function, and suppress inflammatory pathways [29] (Figure 1). However, microbial composition and metabolite production exhibit considerable interindividual variability, which may influence metabolic outcomes. The gut-liver and gut–brain axes, therefore, constitute central regulatory networks in MetS, mediating bidirectional communication between microbial signals and host metabolic pathways. Targeting microbiota composition through dietary and nutraceutical interventions represents a promising yet still evolving therapeutic avenue [30].

## 3. Phytochemical Composition and Nutritional Properties of OFIF

OFIF, commonly known as prickly pear cactus fruits, are cultivated extensively in arid and semi-arid regions and have gained increasing scientific interest due to their complex nutritional and phytochemical profile. Their biochemical composition is influenced by cultivar, pigmentation, ripening stage, agronomic practices, and environmental conditions, contributing to considerable variability in reported values across studies [31]. Such heterogeneity should be carefully considered when comparing compositional data and interpreting biological effects [32]. Importantly, this variability represents a critical limitation for cross-study comparability and translational interpretation, as differences in composition and processing may significantly influence the concentration, bioavailability, and ultimately the reproducibility of observed metabolic outcomes.

OFIF consist predominantly of water (approximately 85–92%), resulting in low energy density [32]. The fresh pulp contains modest carbohydrate levels and negligible lipid content, consistent with hypocaloric dietary patterns [31]. Dietary fiber content ranges between 3 and 5 g per 100 g, largely composed of soluble fractions such as pectin and mucilage [33]. Soluble fibers are known to enhance satiety, attenuate postprandial glycemic excursions, and modulate gut microbiota composition through fermentation and short-chain fatty acid production [33].

**Figure 2:**
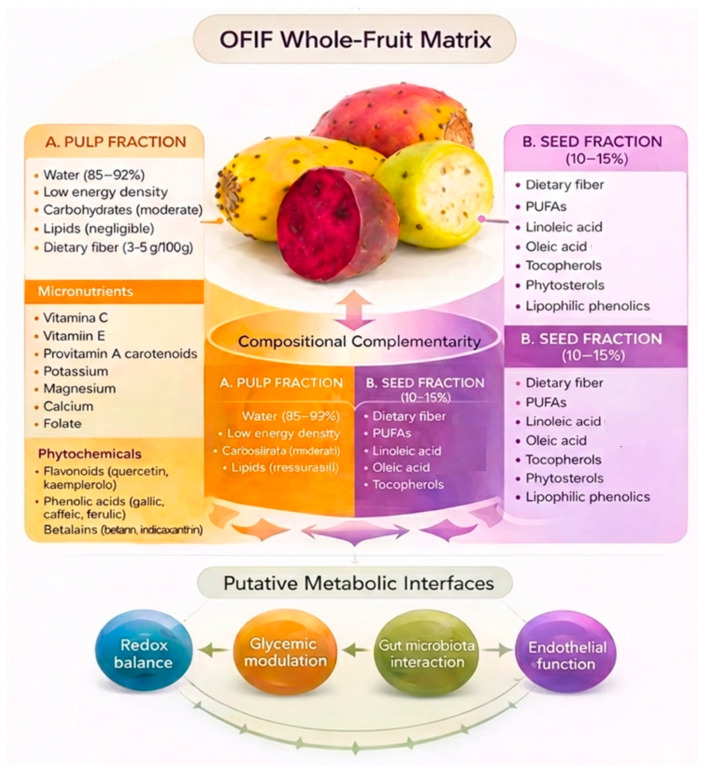
OFIF whole-fruit matrix and compositional complementarity. Schematic representation of the OFIF whole-food matrix highlighting the complementary bioactive composition of pulp and seed fractions. The pulp fraction is characterized by high water content, dietary fiber, vitamins (C, E), carotenoids, minerals, flavonoids, phenolic acids, and betalains, contributing primarily to redox balance and glycemic modulation. The seed fraction (≈10–15% of fresh weight) provides dietary fiber, PUFAs, tocopherols, phytosterols, and lipophilic phenolics, supporting lipid metabolism and endothelial function. The integrated action of these components underlies putative metabolic interfaces involving oxidative balance, glucose regulation, gut microbiota interaction, and vascular health. Abbreviations: OFIF, *Opuntia ficus-indica* fruits; PUFAs, polyunsaturated fatty acids.

From a micronutrient perspective, OFIF provide moderate amounts of antioxidant vitamins, including vitamin C (approximately 10–20 mg/100 g), vitamin E, and provitamin A carotenoids such as β-carotene [32]. They also contain potassium, magnesium, calcium, and folate, with concentrations varying according to cultivar and environmental conditions [31,34]. Although these levels are not pharmacological, the presence of these micronutrients within a fiber- and phytochemical-rich food matrix may contribute to redox balance and metabolic regulation as part of an overall dietary pattern. The phytochemical profile of OFIF is diverse and includes flavonoids such as quercetin, kaempferol, and isorhamnetin derivatives, phenolic acids such as gallic, caffeic, and ferulic acids, and betalains, in particular betanin and indicaxanthin, which confer the characteristic pigmentation [32]. In intensely pigmented cultivars, total betalain concentrations may approach 80–100 mg/100 g fresh weight, although substantial inter-cultivar variation has been reported [35]. Experimental studies demonstrate antioxidant and anti-inflammatory properties of these compounds and suggest potential modulation of metabolic pathways in preclinical models [6,36]. However, the extent to which these mechanistic findings translate to whole-fruit consumption in humans remains to be fully clarified. Notably, OFIF are often consumed whole, including the seeds, which account for approximately 10–15% of total fruit weight [31]. The seeds are enriched in dietary fiber and contain polyunsaturated fatty acids (PUFAs), particularly linoleic acid (ω-6) and oleic acid (ω-9), alongside tocopherols, phytosterols, and lipophilic phenolic compounds [37] (Figure 2). A computational investigation suggested that these constituents exhibit antioxidant and antiglycation properties [38], although clinical confirmation in whole-fruit consumption remains limited. Additionally, seeds make available amino acids such as arginine and glutamic acid, which may contribute to nitric oxide production, endothelial function, and redox balance [39]. Although the quantitative contribution of these amino acids from typical dietary portions requires further evaluation, the combined presence of soluble fibers, bioactive lipids, antioxidant micronutrients, and phenolic compounds highlights a compositional complementarity between pulp and seeds that may support multi-target metabolic modulation [40]. Importantly, the biological effects of OFIF cannot be fully attributed to individual compounds, as the fruit represents a complex phytocomplex in which multiple bioactive components may interact synergistically or antagonistically, thereby influencing bioavailability and overall metabolic impact.

## 4. Polyphenols and Flavonoids from OFIF: Mechanistic Insights into Metabolic Syndrome Modulation

**Figure 3:**
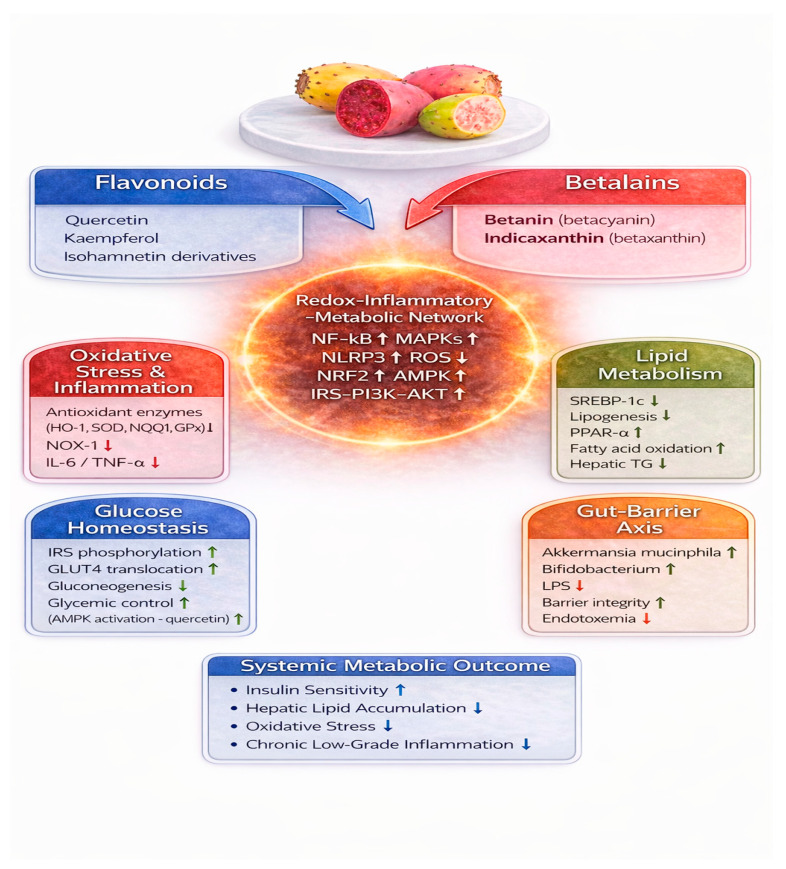
Mechanistic effects of OFIF-derived flavonoids and betalains in metabolic syndrome. This schematic illustrates the principal molecular mechanisms associated with flavonoids (e.g., quercetin, kaempferol, isorhamnetin) and betalains (e.g., betanin, indicaxanthin) derived from *Opuntia ficus-indica* fruits. These bioactive compounds modulate key components of the redox–inflammatory–metabolic network, including activation of NRF2-mediated antioxidant responses, attenuation of NF-κB and NLRP3-dependent inflammatory signaling, and regulation of insulin-related pathways such as IRS-PI3K-AKT and AMPK. Additional effects include modulation of lipid metabolism through SREBP-1c downregulation and PPAR-α activation, as well as potential interactions with gut barrier integrity and microbiota-related signaling. Shared downstream pathways are simplified to highlight compound-specific mechanisms. Most evidence derives from preclinical models, and the extent of translation to human metabolic syndrome remains to be established. Abbreviations: OFIF, *Opuntia ficus-indica* fruits; MetS, metabolic syndrome; NF-κB, nuclear factor kappa B; MAPKs, mitogen-activated protein kinases; NLRP3, NLR family pyrin domain containing 3; ROS, reactive oxygen species; NRF2, nuclear factor erythroid 2–related factor 2; AMPK, AMP-activated protein kinase; IRS, insulin receptor substrate; PI3K, phosphoinositide 3-kinase; AKT, protein kinase B; GLUT4, glucose transporter type 4; SREBP-1c, sterol regulatory element-binding protein 1c; PPAR-α, peroxisome proliferator-activated receptor alpha; LPS, lipopolysaccharide. Upward (↑) and downward (↓) arrows indicate upregulation and downregulation of the indicated processes, respec-tively. Color coding is used to distinguish functional domains: blue denotes flavonoids, red betalains and inflammatory pathways, green lipid metabolism, orange the gut–barrier axis, and light blue glucose homeostasis.

OFIF are rich in polyphenolic compounds, particularly flavonoids such as quercetin, kaempferol, and isorhamnetin derivatives, often present in glycosylated forms [32]. These molecules exhibit antioxidant and anti-inflammatory properties and have been reported to influence glucose and lipid metabolism in experimental models [41]. Mechanistically, flavonoids modulate oxidative stress pathways, insulin signaling cascades, lipid regulatory transcription factors, and intestinal barrier integrity [42,43]. However, it is important to emphasize that much of the mechanistic evidence derives from isolated compounds tested under controlled experimental conditions, which may not fully reflect the complexity of whole-food consumption or physiological exposure levels in humans. Consequently, the direct translation of these findings to clinical settings remains uncertain. Within this context, these mechanisms should be interpreted as components of an integrated redox–inflammatory–metabolic network, as summarized in Figure 3.

### 4.1. Modulation of Oxidative Stress and Inflammation

Flavonoids and other polyphenolic constituents of OFIF have been shown to act as ROS scavengers in experimental systems, attenuating oxidative damage associated with mitochondrial dysfunction in metabolically active tissues [32,44]. In parallel, experimental studies indicate inhibition of pro-inflammatory signaling pathways, including NF-κB and MAPKs, which are upregulated in obesity-related inflammation [6]. In line with the redox–inflammatory axis described above, these compounds inhibit NF-κB and MAPK activation while promoting NRF2 nuclear translocation and antioxidant enzyme expression, thereby contributing to restoration of metabolic homeostasis [18,22] (Figure 3).

### 4.2. Effects on Glucose Metabolism and Insulin Sensitivity

Flavonoids have been reported to modulate the insulin signaling cascade by promoting phosphorylation of IRS-1/2 and activating downstream pathways such as PI3K-AKT, thereby facilitating GLUT4 translocation in skeletal muscle and adipose tissue [45]. Notably, quercetin activates AMPK, a central metabolic regulator that enhances fatty acid oxidation, suppresses hepatic gluconeogenesis, and improves glycemic control in insulin-resistant models [46] (Figure 3).

### 4.3. Regulation of Lipid Metabolism

Experimental studies have shown that flavonoids exert hypolipidemic effects through modulation of key transcriptional regulators of lipid metabolism. Mechanistically, several flavonoid subclasses downregulate sterol regulatory element-binding protein-1c (SREBP-1c), a master regulator of de novo lipogenesis, while upregulating PPAR-α, thereby promoting fatty acid oxidation and reducing hepatic triglyceride accumulation [47]. Through this coordinated transcriptional reprogramming, flavonoids may shift the metabolic balance from lipid synthesis toward lipid utilization [48]. Given that OFIF are enriched in structurally related flavonoid derivatives, it is therefore reasonable to hypothesize that flavonoids present in OFIF may engage analogous lipid-regulatory mechanisms, supporting their proposed role in metabolic modulation (Figure 3).

### 4.4. Gut Microbiota Interaction and Endotoxemia Control

Dietary polyphenols have been shown to modulate gut microbial composition, including selectively increasing the relative abundance of beneficial taxa such as *Akkermansia muciniphila* in both animal and human studies, and are associated with improved metabolic phenotypes in high-fat diet models [49]. Furthermore, grape polyphenol extracts were found to increase *A. muciniphila* abundance and reinforce gut barrier integrity while producing a less inflammatory microbial milieu in experimental systems [50]. These microbiota shifts are hypothesized to reduce circulating endotoxin levels and attenuate low-grade inflammation, a recognized driver of IR in MetS (Figure 3).

## 5. Betalains from *Opuntia ficus-indica*: Mechanistic Insights into Metabolic Syndrome Modulation

Betalains are water-soluble, nitrogen-containing pigments characteristic of the Caryophyllales order, with *OFIF* representing a significant dietary source of betacyanins, including betanin, which is responsible for red-purple pigmentation, and betaxanthins such as indicaxanthin, which is responsible for yellow-orange coloration. These pigments show quantitative variability according to genotype, ripening stage, and post-harvest conditions, which may influence biological exposure after dietary intake [51]. Evidence from in vitro and animal models indicates that betalains can exert antioxidant and anti-inflammatory effects, modulate redox-dependent signaling pathways, and protect vascular cells from oxidative alterations [52] (Figure 3). Despite promising mechanistic findings, the majority of evidence supporting betalain activity is derived from in vitro and animal models. Differences in dosage, formulation, and bioavailability raise important questions regarding the physiological relevance of these effects in humans, particularly in the context of habitual dietary intake.

### 5.1. Modulation of Inflammatory Signaling

Indicaxanthin is one of the few dietary pigments demonstrated to be bioavailable in intact form following oral consumption, with measurable plasma concentrations in humans [52]. Experimental evidence further indicates that indicaxanthin can cross biological barriers, including the blood–brain barrier, and modulate redox status in neural tissues [53]. In vitro and preclinical studies show that indicaxanthin inhibits NF-κB activation and downregulates NADPH oxidase (NOX)-1, resulting in reduced production of pro-inflammatory mediators such as IL-6 and TNF-α [52,54]. Through coordinated modulation of redox and inflammatory pathways, indicaxanthin may therefore influence molecular processes implicated in metabolic dysfunction. However, direct clinical confirmation of these mechanisms in individuals with established MetS remains limited (Figure 3).

### 5.2. Glucose Homeostasis and Redox Enzyme Restoration

Betanin and indicaxanthin have demonstrated antioxidant activity in cellular and animal models. In addition to direct scavenging of reactive oxygen and nitrogen species, betanin has been reported to activate NRF2 signaling and induce NRF2/ARE-regulated cytoprotective enzymes, including HO-1 and NQO1, thereby enhancing endogenous antioxidant defenses [55]. Given the contribution of oxidative stress to mitochondrial dysfunction and IR in MetS, modulation of NRF2 signaling represents a biologically plausible mechanism. However, most evidence supporting this pathway derives from preclinical systems, and its quantitative relevance in human MetS requires further clarification. In experimental models of diet-induced metabolic dysfunction, supplementation with the OFIF indicaxanthin has been associated with improved glycemic control, reduced intrahepatic lipid accumulation, and attenuation of inflammatory and oxidative stress markers, including malondialdehyde [56]. Complementary preclinical data also indicate reductions in MDA and restoration of antioxidant enzyme activity, such as SOD and GPx, following betanin intake [57]. While these findings strengthen biological plausibility, dose equivalence between experimental models and typical human consumption patterns remains to be defined (Figure 3).

### 5.3. Hepatic Lipid Metabolism and NAFLD

Preclinical studies using high-fat diet (HFD)-induced models of metabolic dysfunction have shown that supplementation with betalain-rich OFIF extracts or purified indicaxanthin attenuates hepatic lipid accumulation and improves histological features of steatosis [56]. In experimental settings, these metabolic improvements have been associated with modulation of lipid-regulatory pathways implicated in de novo lipogenesis and fatty acid oxidation. Although some studies suggest downregulation of lipogenic mediators and enhanced oxidative metabolism, direct and consistent demonstration of specific transcriptional targets such as SREBP-1c, FAS, PPAR-α, or CPT1α in OFIF-specific models remains limited. Variability in extract composition, betalain standardization, and dosage across studies could further provide a mechanistic rationale for their observed metabolic effects in preclinical models (Figure 3).

### 5.4. Endothelial Protection and Vascular Inflammation

In endothelial cell models, indicaxanthin has been shown to attenuate inflammatory activation by reducing the expression of adhesion molecules such as ICAM-1 and, in inflammatory conditions, modulating related vascular inflammatory pathways [54,58] (Figure 3). These effects are associated with modulation of redox-dependent signaling cascades implicated in early atherogenic processes [54]. While these mechanistic findings are consistent with potential cardiometabolic protection, translational studies assessing vascular endpoints in individuals with established MetS remain limited.

### 5.5. Gut Microbiota Modulation

Emerging evidence from experimental models indicates that dietary polyphenols, including betalain-rich plant extracts, may modulate gut microbial ecology by increasing the relative abundance of beneficial taxa such as *Akkermansia muciniphila* and *Bifidobacterium* spp., while reducing pro-inflammatory Gram-negative bacteria [51,59] (Figure 3). These microbiota shifts have been associated with improved intestinal barrier integrity and reduced metabolic endotoxemia in high-fat diet models [60]. Although such findings provide a mechanistic rationale linking betalain-rich dietary interventions to metabolic regulation, direct clinical evidence in individuals with established MetS remains limited. Given the substantial interindividual variability in gut microbiota composition, further well-controlled studies are required to determine the reproducibility and durability of these effects across diverse populations.

## 6. Carotenoids from *Opuntia ficus-indica*: Molecular Targets in Metabolic Syndrome

**Figure 4:**
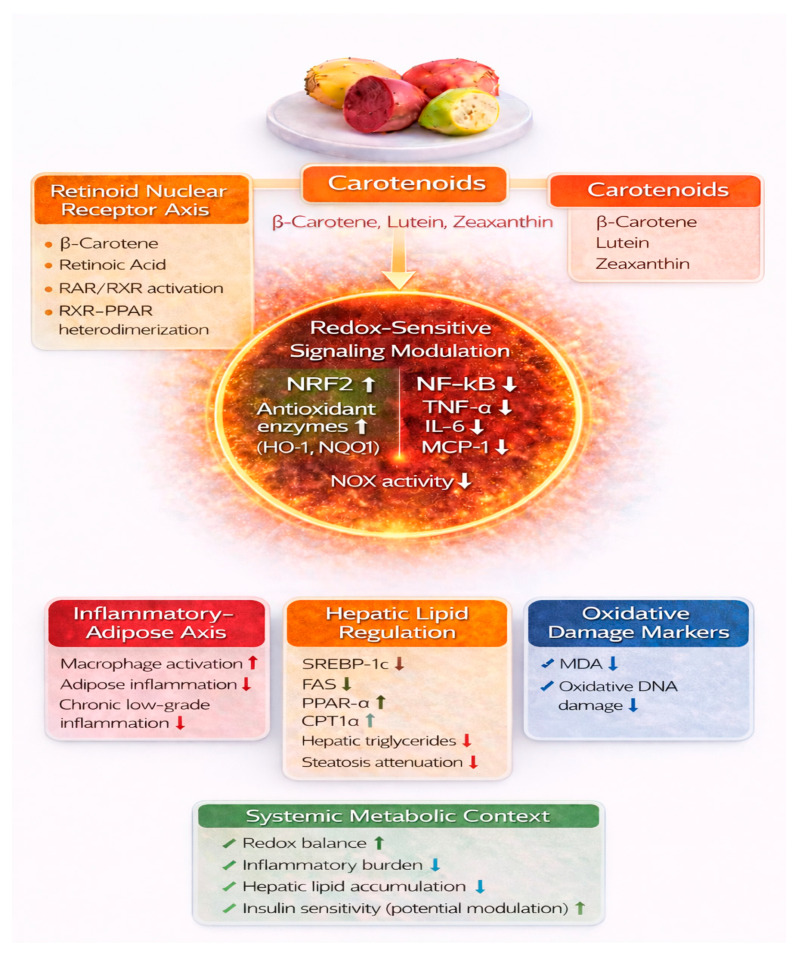
Molecular pathways modulated by OFIF-derived carotenoids in metabolic syndrome. This schematic focuses on the mechanisms associated with carotenoids (e.g., β-carotene, lutein, zeaxanthin) present in *Opuntia ficus-indica* fruits. Carotenoids exert antioxidant and immunomodulatory effects through activation of nuclear receptor signaling pathways, including RAR/RXR and their interaction with PPAR isoforms, thereby influencing transcriptional regulation of lipid and glucose metabolism. These compounds contribute to the enhancement of NRF2-mediated antioxidant defenses and attenuation of NF-κB-driven inflammatory signaling, while also modulating lipid homeostasis (↓ SREBP-1c, ↓ FAS, ↑ PPAR-α, ↑ CPT1α) and oxidative stress markers. Although partially overlapping with other OFIF bioactives in downstream signaling, this figure emphasizes carotenoid-specific regulatory pathways and nuclear receptor–mediated effects. Current evidence is largely derived from experimental and observational studies, with limited direct clinical validation in metabolic syndrome. Upward (↑) and downward (↓) arrows indicate activation/upregulation and inhibition/downregulation of the indicated pathways, respectively. Color coding is used to distinguish functional domains: orange denotes carotenoid-related pathways, red inflammatory signaling, blue oxidative damage markers, and green systemic metabolic effects. Abbreviations: NRF2, nuclear factor erythroid 2–related factor 2; NF-κB, nuclear factor kappa B; TNF-α, tumor necrosis factor alpha; IL-6, interleukin-6; MCP-1, monocyte chemoat-tractant protein-1; NOX, NADPH oxidase; HO-1, heme oxygenase-1; NQO1, NAD(P)H quinone dehydrogenase 1; SREBP-1c, ster-ol regulatory element-binding protein 1c; FAS, fatty acid synthase; PPAR-α, peroxisome proliferator-activated receptor alpha; CPT1a, carnitine palmitoyltransferase 1a; MDA, malondialdehyde.

Carotenoids are lipophilic pigments widely recognized for their antioxidant and immunomodulatory properties. Orange- and red-fleshed OFIF contain provitamin A carotenoids, including β-carotene, as well as xanthophylls such as lutein and zeaxanthin [32]. Although their concentrations in OFIF are moderate compared with highly carotenoid-dense fruits and vegetables, their presence within a bioactive-rich matrix may contribute to integrated metabolic responses associated with whole-fruit consumption. In this context, isolating the specific contribution of carotenoids is inherently challenging, as synergistic or additive interactions within the food matrix likely influence biological outcomes [61] (Figure 4). While flavonoids and betalains predominantly act on the central redox–inflammatory–metabolic axis (Figure 3), carotenoids engage complementary regulatory mechanisms, including RAR/RXR- and PPAR-mediated transcriptional pathways (Figure 4). Although partial overlap exists at the level of downstream signaling, these bioactive classes contribute distinct yet integrated layers of metabolic regulation within the complex pathophysiology of metabolic syndrome. The contribution of carotenoids from OFIF must be interpreted within the broader dietary context, as their metabolic effects are likely influenced by overall nutritional patterns, bioavailability, and interactions with other bioactive compounds within the food matrix.

Beyond direct radical scavenging, carotenoids modulate redox-sensitive signaling pathways involved in inflammatory and metabolic regulation. Experimental studies indicate that selected carotenoids can enhance NRF2 activation and promote transcription of antioxidant defense enzymes, while attenuating NF-κB–dependent inflammatory signaling under conditions of oxidative or metabolic stress [62].

In human observational studies, higher dietary carotenoid intake has been associated with reduced biomarkers of lipid peroxidation and oxidative DNA damage (Table 1). For instance, carotenoid consumption was inversely related to circulating malondialdehyde levels and DNA damage markers in middle-aged men [63]. Although these findings do not establish causality, they support the potential contribution of carotenoid-rich foods to systemic redox balance within a dietary context.

### 6.1. Modulation of Inflammatory Signaling

Experimental evidence indicates that lutein and zeaxanthin attenuate inflammatory signaling in multiple cell types relevant to metabolic dysfunction. In macrophages and adipocytes, lutein suppresses NF-κB activation and reduces the expression of pro-inflammatory mediators, including TNF-α, IL-6, and MCP-1 [64]. Similarly, zeaxanthin has been shown to modulate redox-sensitive inflammatory pathways in endothelial cells, contributing to reduced NF-κB–dependent signaling [65]. By attenuating chronic low-grade inflammation, carotenoids may therefore influence one of the central pathogenic links between visceral adiposity and IR [62] (Figure 4).

### 6.2. Regulation of Lipid Metabolism

Carotenoids and their bioactive metabolites have been shown to influence transcriptional regulators of lipid metabolism in experimental systems. β-Carotene-derived retinoic acid can suppress lipogenic gene expression, including SREBP-1c and fatty acid synthase (FAS), while promoting pathways associated with fatty acid oxidation through modulation of PPAR-α and CPT1α signaling [66]. In preclinical models of diet-induced metabolic dysfunction, carotenoid-rich interventions have been associated with attenuation of hepatic steatosis and modulation of hepatic lipid metabolism. For example, carotenoids derived from orange carrots mitigated NAFLD progression and reduced hepatic inflammatory and oxidative stress markers in murine models [67] (Figure 4).

Beyond these lipid-modulating effects, β-carotene functions as a precursor of retinoic acid, a transcriptionally active metabolite that binds retinoic acid receptors (RARs) and retinoid X receptors (RXRs). Through RXR heterodimerization with PPAR isoforms and other nuclear receptors, retinoid signaling integrates pathways governing adipogenesis, lipid handling, and glucose homeostasis [68]. This nuclear receptor cross-talk provides a mechanistic framework linking carotenoid intake to broader metabolic gene regulation. Nevertheless, the quantitative contribution of β-carotene derived specifically from OFIF to systemic retinoid signaling in human MetS remains to be clarified.

## 7. Effects of Vitamins and Minerals in Metabolic Syndrome

Beyond their phytochemical constituents, OFIF provide a spectrum of micronutrients that may contribute to metabolic regulation and vascular homeostasis. Compositional analyses indicate the presence of antioxidant vitamins such as vitamin C, vitamin E [31,32] and provitamin A, which was discussed at length in the previous section.

Vitamin C and vitamin E represent key components of endogenous antioxidant defense systems. Vitamin C contributes to the regeneration of oxidized α-tocopherol and directly scavenges reactive oxygen species, whereas vitamin E protects membrane polyunsaturated fatty acids from lipid peroxidation [69,70]. In cardiometabolic contexts characterized by increased oxidative stress and endothelial dysfunction, adequate dietary intake of antioxidant vitamins has been associated with improvements in endothelial reactivity and reductions in oxidative biomarkers in selected populations [71]. However, large-scale randomized trials using high-dose antioxidant supplementation have yielded heterogeneous or neutral outcomes, underscoring the distinction between physiological intake within a complex food matrix and pharmacological supplementation [72].

Although the concentrations of these micronutrients in OFIF are moderate compared with pharmacological doses, their coexistence within a fiber- and phytochemical-rich matrix may support additive or synergistic metabolic effects consistent with the concept of food synergy [73]. Nevertheless, the quantitative contribution of OFIF consumption alone to micronutrient status and cardiometabolic outcomes in individuals with established metabolic syndrome remains to be defined in controlled clinical settings.

### Mineral Contributions to Glycemic and Vascular Regulation

OFIF provide potassium and magnesium in nutritionally relevant amounts, along with trace elements such as calcium and zinc, although concentrations vary according to cultivar, environmental conditions, and post-harvest handling [74]. Within habitual dietary patterns, these minerals contribute to glucose regulation, vascular homeostasis, and inflammatory balance.

Magnesium plays a central role in insulin receptor activity, glucose transport, and ATP-dependent enzymatic reactions involved in carbohydrate metabolism. Suboptimal magnesium status has been consistently associated with IR and increased cardiometabolic risk, while supplementation in deficient individuals has been linked to improvements in insulin sensitivity and endothelial function [75,76]. Magnesium also modulates inflammatory mediators, including C-reactive protein, IL-6, and TNF-α, further linking mineral status to low-grade systemic inflammation [77].

**Figure 5:**
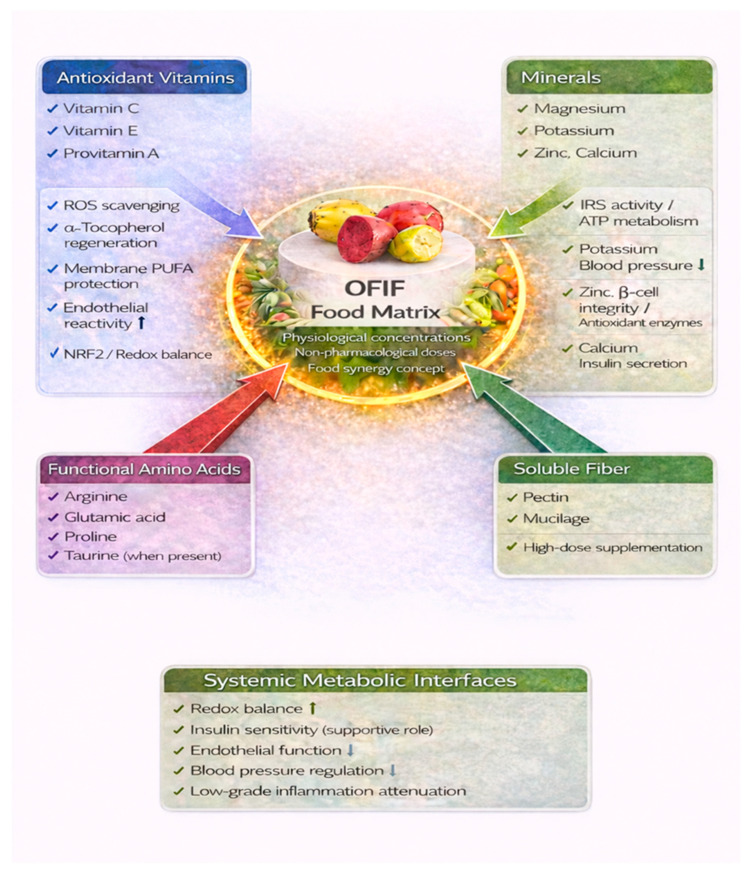
Integrated contribution of micronutrients within the OFIF whole-food matrix in metabolic syndrome. This schematic highlights the role of vitamins (e.g., vitamin C, vitamin E, provitamin A carotenoids) and minerals (e.g., magnesium, potassium, zinc, calcium) present in OFIF as components of a complex nutritional matrix. Unlike Figure 3 and Figure 4, which focus on bioactive-driven intracellular signaling pathways, this figure emphasizes food-level interactions and micronutrient-mediated support of metabolic and vascular homeostasis, including antioxidant defense, insulin signaling support, endothelial function, and blood pressure regulation. Shared downstream effects with other bioactive compounds are simplified to underscore the concept of food synergy rather than pathway redundancy. Abbreviations: OFIF, *Opuntia ficus-indica* fruits; ROS, reactive oxygen species; NRF2, nuclear factor erythroid 2–related factor 2; IRS, insulin receptor substrate; ATP, adenosine triphosphate; PUFA, polyunsaturated fatty acid. Arrows indicate the direction of interaction and functional contribution of each component to the OFIF food matrix. Color coding distinguishes nutrient classes: blue denotes antioxidant vitamins, green minerals and systemic metabolic interfaces, purple functional amino acids, and light green soluble fiber. The central concept highlights the synergistic action of whole-food components at physiological, non-pharmacological concentrations.

Potassium intake is inversely associated with blood pressure and cardiovascular risk, with meta-analytic evidence supporting its role in vascular function and stroke reduction [78,79]. Zinc contributes to pancreatic β-cell integrity, insulin synthesis and storage, and antioxidant enzyme activity, supporting glycemic control and redox homeostasis [80]. Calcium participates in insulin secretion and intracellular signaling processes that regulate metabolic responsiveness [81] (Figure 5).

Although the mineral content of OFIF does not reach pharmacological levels, their coexistence within a fiber and phytochemical-rich matrix may contribute to cumulative metabolic support through additive or synergistic interactions. Nevertheless, the extent to which OFIF consumption alone meaningfully modifies micronutrient status in individuals with established metabolic syndrome remains to be quantitatively defined in controlled clinical trials.

## 8. Functional Amino Acids and Soluble Fiber: Integrated Metabolic Modulation

Beyond micronutrients and classical phytochemicals, OFIF contain bioactive amino acids and soluble fibers that may contribute to metabolic regulation. Amino acid profiling studies have identified arginine, proline, glutamic acid, and other free amino acids in cactus pear fruits, with quantitative variability across cultivars [31]. Taurine, when present, has been shown in experimental systems to modulate endoplasmic reticulum stress, mitochondrial function, and inflammatory signaling pathways linked to IR and hepatic steatosis [82]. Arginine serves as a substrate for nitric oxide synthesis via endothelial nitric oxide synthase, thereby influencing vascular tone and insulin-mediated glucose uptake [83]. However, the amounts obtainable through typical OFIF consumption remain substantially lower than those used in supplementation studies.

Soluble fibers, particularly pectin and mucilage, constitute a more quantitatively relevant component of the fruit matrix. By increasing luminal viscosity, soluble polysaccharides delay gastric emptying and attenuate postprandial glycemic excursions [84]. Fermentation by gut microbiota produces SCFAs, including butyrate and propionate, which regulate epithelial barrier integrity, hepatic gluconeogenesis, lipid oxidation, and inflammatory signaling pathways relevant to the gut–liver axis in metabolic syndrome [85] (Figure 5). Taken together, the coexistence of functional amino acids, fermentable fibers, micronutrients, and phytochemicals within OFIF supports a multi-component dietary framework in which complementary mechanisms may operate simultaneously. However, standardized human intervention studies are required to quantify the magnitude and reproducibility of these effects in established MetS populations.

## 9. Evidence from Animal and Human Studies on OFIF in Metabolic Syndrome

Multiple in vivo studies have investigated OFIF supplementation in high-fat-diet (HFD)-induced models of metabolic dysfunction. Collectively, these studies report attenuation of body weight gain, visceral adiposity, serum triglycerides, and hepatic steatosis following administration of whole-fruit extracts or purified fractions [6,57]. Improvements in glycemic control, oxidative stress parameters, and partial restoration of gut microbiota composition have also been described in experimental settings [86].

Rather than demonstrating isolated effects on single pathways, these models suggest a coordinated impact on hepatic lipid handling, systemic redox balance, and adipose tissue inflammation. In some studies, betalain-enriched preparations have been associated with enhanced hepatic fatty acid oxidation and modulation of lipogenic mediators, although mechanistic attribution to specific phytochemical classes remains limited by variability in extract composition and standardization.

Direct comparisons with pharmacological agents should be interpreted cautiously, as experimental paradigms differ substantially in dosage, duration, and severity of metabolic induction. Furthermore, dose equivalence between animal models and achievable human dietary intake has not been systematically established. Taken together, preclinical evidence supports biological plausibility for OFIF-mediated metabolic modulation, yet heterogeneity in animal models, extract preparation, and outcome measures limits cross-study comparability and precludes robust quantitative synthesis. Rigorous human intervention trials with standardized compositional characterization are therefore required to determine translational relevance in established MetS populations (Table 1).

Human studies investigating OFIF remain limited in number, duration, and sample size but provide preliminary indications of biological activity. Short-term dietary interventions in healthy volunteers have reported reductions in oxidative stress markers and lipid peroxidation following OFIF consumption [35]. Early investigations of betalain-rich cultivars similarly documented decreases in circulating oxidative biomarkers, although inflammatory endpoints were variably assessed. Pharmacokinetic studies have confirmed that indicaxanthin is bioavailable in humans at nutritionally relevant doses, supporting the plausibility of systemic effects [44] (Table 1). However, controlled trials specifically targeting individuals with established metabolic syndrome are scarce. Available pilot studies have generally involved small cohorts, short intervention periods, and heterogeneous fruit preparations, limiting causal inference and generalizability. At present, human evidence supports safety and mechanistic plausibility but remains insufficient to establish definitive clinical efficacy in MetS populations. Future investigations should employ adequately powered randomized controlled designs, standardized compositional characterization of OFIF preparations (including quantified betalain, fiber, and carotenoid content), and clearly defined metabolic endpoints such as insulin sensitivity indices, hepatic fat quantification, endothelial function, and inflammatory biomarkers.

## 10. Safety Considerations and Potential Limitations

Although OFIF are generally considered safe as part of a traditional diet, potential limitations and adverse effects should be acknowledged. High intake of fiber-rich fractions may lead to gastrointestinal discomfort, including bloating or altered bowel habits, particularly in sensitive individuals. Moreover, the hypoglycemic potential of OFIF-derived compounds raises the possibility of additive effects in individuals receiving glucose-lowering therapies, warranting cautious interpretation in clinical contexts. Variability in bioactive composition across preparations further complicates safety assessment, particularly when extracts or concentrated formulations are used. At present, robust long-term safety data in individuals with established metabolic syndrome remain limited.

## 11. Conclusions and Future Directions

Metabolic syndrome represents a systems-level cardiometabolic condition characterized by intertwined disturbances in insulin signaling, lipid handling, oxidative stress, inflammatory activation, endothelial function, and gut–host communication. Within this pleiotropic regulatory landscape multidimensional framework, OFIF offer a biologically coordinated phytochemical network containing betalains, carotenoids, polyphenols, soluble fibers, functional amino acids, and essential micronutrients. Collectively, these constituents converge on central metabolic pathways, including redox-sensitive pathways NRF2, inflammatory mediators such as NF-κB, energy-sensing regulators such as AMPK, lipid transcription networks such as PPAR-dependent mechanisms, nitric oxide signaling, and microbiota-derived metabolic circuits, highlighting a coordinated, systems-level mode of action rather than isolated pharmacological effects.

Despite compelling mechanistic plausibility and consistent trends across preclinical models, translational limitations remain substantial. Experimental systems frequently employ doses, extract preparations, and metabolic contexts that differ from habitual human consumption. Clinical investigations to date have been limited by modest sample sizes, short intervention periods, and heterogeneity in cultivar composition and product standardization. Furthermore, most available studies involve healthy or mildly at-risk individuals, leaving the efficacy of OFIF in established MetS, T2D, or NAFLD populations insufficiently defined.

Future research should prioritize rigorously standardized OFIF preparations with detailed compositional profiling, adequately powered randomized controlled trials in well-characterized cardiometabolic cohorts, and integration of mechanistic endpoints, including insulin sensitivity indices, hepatic fat quantification, endothelial function assessment, inflammatory mediators, and gut microbiota signatures. Clarifying dose–response relationships within achievable dietary ranges will be essential to determine real-world applicability. This review highlights the need to move beyond descriptive associations toward rigorously designed human studies, including standardized OFIF preparations, dose–response assessments, and clinically meaningful endpoints. Future research should further clarify bioavailability, interindividual variability (including microbiota-dependent responses), and long-term safety. From a translational perspective, OFIF should be interpreted not as a pharmacological intervention, but as a component of a broader dietary framework in which synergistic interactions within the food matrix may contribute to metabolic resilience. Importantly, future investigations should move beyond reductionist approaches focused on isolated compounds and instead prioritize the study of whole-food matrices, where interactions among bioactive components are likely to determine the net biological effect.

## Figures and Tables

**Table 1:** Preclinical and Human Evidence on OFIF in Metabolic Syndrome: Mechanistic Targets and Translational Relevance.

Study Type	Model/Population	OFIF Preparation	Main Metabolic Outcomes	Molecular Targets/Pathways	Key References
Preclinical (in vivo)	HFD-induced metabolic dysfunction (rodents)	Whole-fruit extracts	↓ Body weight gain; ↓ Visceral adiposity; ↓ Serum TG; ↓ Hepatic steatosis	SREBP-1c ↓; Lipogenic mediators ↓; Fatty acid oxidation ↑; Oxidative stress markers ↓	[6,57]
Preclinical (betalain-enriched)	HFD rodent models	Betalain-rich fractions/purified indicaxanthin	Improved glycemic control; ↓ Intrahepatic lipid accumulation; ↓ MDA	NRF2 activation ↑; HO-1 ↑; NQO1 ↑; NF-κB ↓; NOX-1 ↓	[52,55,56,57]
Preclinical (gut axis)	Diet-induced metabolic dysfunction	Whole-fruit extracts	Partial restoration of gut microbiota; ↓ Inflammation	SCFA-related pathways; Barrier integrity ↑; LPS signaling ↓	[57]
Preclinical (lipid metabolism focus)	HFD models	Variable OFIF preparations	Attenuation of NAFLD features	PPAR-α ↑; CPT1α ↑ (suggested); SREBP-1c ↓ (inconsistent evidence)	[56,57]
Human (acute intervention)	Healthy volunteers	Fresh OFIF consumption	↓ Lipid peroxidation; ↓ Oxidative biomarkers	Systemic redox modulation; indirect NRF2-related antioxidant activity (associative)	[36]
Human (pharmacokinetic)	Healthy subjects	Purified indicaxanthin	Demonstrated plasma bioavailability	Circulating intact indicaxanthin; supporting redox pathway interaction	[44]
Human (pilot metabolic studies)	Small cohorts	Betalain-rich cultivars	↓ Oxidative biomarkers; variable inflammatory endpoints	NF-κB-related inflammatory modulation (indirect evidence)	[36]
Human (MetS-specific)	Established MetS	Limited controlled trials	Insufficient evidence for clinical efficacy	Target pathways hypothesized (NRF2, NF-κB, lipid regulators), not directly validated in vivo	[44]

Level of evidence reflects the degree of translational validation from preclinical models to human intervention studies. Across studies, several limitations should be considered, including small sample sizes, short intervention duration, heterogeneity in OFIF preparation, lack of standardized dosing, limited availability of randomized controlled trials, and a predominant reliance on surrogate biomarkers rather than clinically meaningful endpoints. Abbreviations: HFD, high-fat diet; TG, triglycerides; MDA, malondialdehyde; SCFA, short-chain fatty acids; NRF2, nuclear factor erythroid 2–related factor 2; NF-κB, nuclear factor kappa B; NOX-1, NADPH oxidase-1.

## Data Availability

The original contributions presented in this study are included in the article. Further inquiries can be directed to the corresponding author.
